# Examining Pediatric Emergency Utilization Trends Before and After the COVID-19 Pandemic: An Eight-Year Cohort Study from a South Korean Tertiary Center

**DOI:** 10.3390/children12091232

**Published:** 2025-09-15

**Authors:** Hae Jeong Lee, Yechan Kyung, Dong Wan Kang, Mi Hyeon Jin, Seoheui Choi, Jun Hwa Lee

**Affiliations:** 1 Department of Pediatrics, Samsung Changwon Hospital, Sungkyunkwan University School of Medicine, Changwon 51353, Republic of Korea; okii1004@hanmail.net (H.J.L.); drchany@skku.edu (Y.K.); eugenestyle.kang@samsung.com (D.W.K.); 2 Department of Research & Support, Samsung Changwon Hospital, Sungkyunkwan University School of Medicine, Changwon 51353, Republic of Korea; mihyeon.jin@samsung.com; 3 Department of Pediatrics, Ajou University School of Medicine, Suwon 16499, Republic of Korea; shchoi@aumc.ac.kr

**Keywords:** pediatrics, emergency department, COVID-19, trend, Korea

## Abstract

**Purpose:** This study investigates trends in pediatric emergency department (ED) utilization before and after the COVID-19 pandemic, with a focus on age-specific patterns, triage severity, diagnostic categories, and clinical presentations. **Methods:** Data were collected for 71,560 individuals (40,428 males and 31,132 females aged 0–18 years) who visited the ED at Samsung Changwon Hospital between 1 January 2016 and 31 December 2023. Patients were categorized into pre-COVID-19 (2016–2019) and post-COVID-19 (2020–2023) periods. Age, Korean Triage and Acuity Scale (KTAS) scores, visit outcomes, diagnostic codes (ICD-10), and vital signs were analyzed. Age-specific analyses were performed in four groups: <12 months, 1–6 years, 7–12 years, and 13–18 years. **Results:** Since the COVID-19 pandemic, pediatric ED visits have decreased by 55.5%. The proportion of visits by infants (<12 months) and young children (1–6 years) decreased, and adolescent visits increased. Post-pandemic, there was a significant increase in lower-acuity visits (KTAS 4) and discharge rates, alongside a reduction in admissions. Visits for respiratory and infectious diseases (ICD-10 J and A & B codes) decreased markedly, and visits for non-specific symptoms (R codes) and trauma (S & T codes) increased. The mean body weight of young children increased significantly after the COVID-19 period. **Conclusions:** The COVID-19 pandemic has had a profound and lasting effect on pediatric emergency department utilization, with changes in the number of visits, illness patterns, and severity by age group. These findings highlight the need for age-specific strategies in emergency planning and pediatric public health policy, particularly in managing the indirect effects of pandemic-induced changes in behavior and access to healthcare.

## 1. Introduction

In January 2020, coronavirus disease 2019 (COVID-19) was first identified in Wuhan, China, and spread worldwide. The COVID-19 pandemic has had major effects on healthcare systems and pediatric emergency care, causing notable shifts [1,2]. This unprecedented public health crisis has led to noticeable changes in pediatric emergency department utilization patterns [2,3]. During the COVID-19 pandemic, a combination of factors, including concerns about exposure to the virus, lockdowns, and public health communications, led many families to delay or avoid seeking medical care for mild symptoms [4,5]. Those changes have resulted in a significant overall decline in pediatric emergency department (ED) visits and an increase in the severity of presenting cases [2,5]. Understanding these changes is essential for optimizing resource allocation, enhancing preparedness, and improving patient outcomes in future public health emergencies [2,3,5]. Previous studies have documented the immediate effects of the pandemic on pediatric ED utilization, but most of those were limited to short-term assessments focused on visit volume and diagnostic patterns [3,6]. In this study, we go beyond those immediate effects to elucidate lasting alterations in healthcare utilization behavior and clinical presentation. Through an extensive analysis spanning eight years (2016 to 2023), we identify ongoing changes that might continue to alter pediatric emergency care in the post-pandemic era. Specifically, we use a long-term analysis to examine trends in pediatric emergency department visits pre- and post-COVID-19-pandemic and analyze changes in patient demographics, clinical patterns, and healthcare utilization. This analysis provides valuable insights that can inform future policy and practice in pediatric emergency care and contribute to building healthcare systems that can be more resilient and responsive to future public health emergencies.

## 2. Materials and Methods

### 2.1. Study Population and Study Design

Samsung Changwon Hospital, a tertiary general hospital on the southeastern coast of South Korea, is in Changwon-si, a self-governing city in Gyeongsangnam-do with a population of approximately one million and a total area of 748.03 km^2^ [7]. As of 2021, the pediatric population (younger than 18 years) in Changwon accounted for approximately 15.2% (*n* = 156,895) of the city’s total population [8]. We conducted a retrospective analysis to examine pediatric ED visits at Samsung Changwon Hospital from January 2016 through December 2023. We reviewed the electronic medical records of 71,560 patients younger than 19 years (40,428 males and 31,132 females) who presented to the ED during the study period.

Our investigation focused on temporal trends and patterns in ED utilization before and during the COVID-19 pandemic. We collected comprehensive data on patient demographics, clinical presentations, and outcomes. Specifically, we analyzed sex, age group (<12 months, 1–6 years, 7–12 years, 13–18 years), Korean Triage and Acuity Scale (KTAS) scores, visit dispositions, mode of arrival, timing of visits (day of week, year, month, time of day), influenza and COVID-19 test results, vital signs, and body weight. KTAS is a patient severity and urgency classification tool developed in 2012 by the Korean Society of Emergency Medicine through an agreement with the Canadian Society of Emergency Medicine to modify the Canadian Triage and Acuity Scale to fit the Korean emergency care system, and consists of a five-level system ranging from 1 (most urgent) to 5 (least urgent), with levels 1–3 considered high-acuity [9].

To assess pandemic-related changes, we divided the study period into pre-pandemic (January 2016–December 2019) and post-pandemic (January 2020–December 2023) phases.

### 2.2. Statistical Analysis

All statistical analyses were conducted using Stata 15.1 (Stata Corporation, College Station, TX, USA). Descriptive statistics are reported as the mean and standard deviation for numeric variables and the frequency and percentage values for categorical variables. Independent *t*-tests, Pearson’s chi-squared test, and Fisher’s exact test were used to compare differences between the two time periods. A statistically significant difference was defined as a *p*-value < 0.05.

### 2.3. Ethics Statement

The study protocol received approval from the Institutional Review Board (IRB) at Samsung Changwon Hospital in Changwon, Korea (IRB No. SCMC 2024-05-003). All data were anonymized prior to analysis, and no identifiable personal information was used.

Given the retrospective design and our use of de-identified data, the requirement for informed consent was waived by the IRB in accordance with institutional guidelines and the Declaration of Helsinki.

## 3. Results

### 3.1. Demographic and Clinical Characteristics Before and After the COVID-19 Pandemic

We analyzed 71,560 pediatric ED visits, comprising 49,526 visits in the pre-COVID-19 period (2016–2019) and 22,034 visits in the post-COVID-19 period (2020–2023). The number of ED visits decreased by 55.5% after the onset of COVID-19. Table 1 compares the demographic and clinical characteristics of pediatric ED visits in the two periods. The age distribution of pediatric ED visits changed significantly after the COVID-19 pandemic (*p* < 0.001).

Following the pandemic, the proportion of younger patients, particularly those younger than 12 months and those 1–6 years of age, decreased, and the proportion of older children and adolescents (7–18 years) increased.

The distribution of KTAS scores changed significantly (*p* < 0.001), with a slight increase in the highest-acuity cases (KTAS 1: 0.43% to 0.74%), an increase in KTAS 4 cases (46.97% to 55.85%), and a decrease in KTAS 2 and 3 cases. The proportion of patients discharged to home increased from 81.49% to 86.41%, and admissions decreased from 18.25% to 12.94% (*p* < 0.001). Transfers and deaths were rare, but their proportion did increase slightly.

Ambulance utilization increased significantly from 6.10% to 10.31% (*p* < 0.001). There was a shift toward more weekday visits (51.24% to 54.63%, *p* < 0.001) in the post-COVID-19 period.

No significant change was observed in daytime versus nighttime visit patterns (*p* = 0.084).

The prevalence of influenza cases decreased significantly, particularly influenza B (6.16% to 1.38%, *p* < 0.001). After the onset of the pandemic, 9351 COVID-19 tests were performed, with 7.89% positive results and 0.48% borderline results.

The mean body temperature and respiratory rate showed slight but statistically significant decreases (37.71 °C to 37.57 °C, *p* < 0.001; 27.64 to 23.75 breaths/min, *p* < 0.001). Age-stratified analysis of respiratory rate (RR) confirmed expected physiological differences, with infants demonstrating the highest baseline RR, followed by a gradual decline with increasing age. All age groups showed a significant reduction in RR after the onset of COVID-19.

The average body weight of the patients increased significantly (19.81 kg to 26.48 kg, *p* < 0.001), with notable increases in the 1–6-years and 7–13-years age groups (*p* < 0.001 for both). When stratified by two-year age intervals, significant increases in body weight were observed in children aged 3–4, 7–8, 9–10, 11–12, and 13–14 years, whereas infants and older adolescents showed no significant changes.

**Figure 1:**
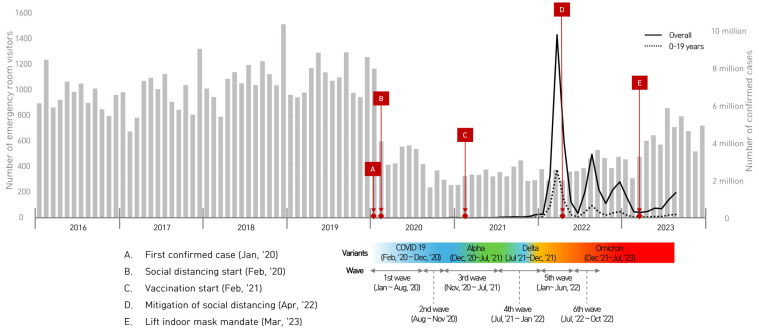
Monthly trends in pediatric emergency department (ED) visits at a single tertiary hospital in Korea from 2016 to 2023. Gray bars (left *Y*-axis) represent the number of ED visits by children and adolescents aged 0–18 years. Overlaid lines (right *Y*-axis) show national trends in confirmed COVID-19 cases: solid line for the total population and dotted line for individuals aged 0–19 years. Key public health events and policy changes related to the COVID-19 pandemic are marked with red diamonds (A–E), and the progression of viral variants and pandemic waves is displayed at the bottom of the chart. COVID-19 incidence data were obtained from the Korea Disease Control and Prevention Agency (KDCA) Infectious Disease Portal [10].

Figure 1 shows a marked decline in pediatric ED visits beginning in early 2020, following the first confirmed COVID-19 case and implementation of social distancing measures. Compared with the pre-pandemic period (2016–2019), ED utilization during the pandemic (2020–2023) remained substantially lower, particularly during periods of intense public health restrictions.

Key national events are annotated: the first confirmed case (January 2020), start of social distancing (February 2020), start of vaccination (February 2021), easing of distancing measures (April 2022), and lifting of indoor mask mandates (March 2023). The lower panel denotes the dominant SARS-CoV-2 variants (Alpha, Delta, Omicron) and Korea’s six major epidemic waves.

### 3.2. Distribution of Diagnoses Based on ICD-10 Categories

The distribution of diagnoses based on ICD-10 categories differed with statistical significance between the two periods (*p* < 0.001), as summarized in Table 2.

The most frequently recorded diagnostic category was R (symptoms, signs, and abnormal clinical findings), which exhibited a marked increase in proportion after the onset of COVID-19, rising from 36.4% to 55.3%. This was followed by S & T codes (injury, trauma, and poisoning), which also demonstrated a notable increase (from 16.5% to 22.5%). In contrast, J codes (respiratory diseases) and A & B codes (infectious and parasitic diseases) declined sharply, from 21.3% to 6.0% and 10.0% to 1.9%, respectively, after the pandemic. Using those detailed shifts, Table 3 provides an overall comparison between medical-disease- and trauma-related visits before and after COVID-19. The proportion of visits due to medical diseases decreased significantly from 83.53% before the pandemic to 77.53% after the pandemic (*p* < 0.001). Conversely, visits related to trauma, injury, or poisoning increased from 16.47% before COVID-19 to 22.47% after COVID-19. Trauma and injury patterns showed distinct age-specific changes before and after the COVID-19 pandemic (Table S1). Head injuries (S06.0) increased across all age groups, particularly among infants and school-aged children. Foreign body ingestion (T17 codes) and upper limb injuries (S53 codes) were more frequently observed in preschool children, whereas musculoskeletal injuries such as ankle sprains (S93.49) and drug-related injuries (T42.7) became more prominent among adolescents.

Table 4 demonstrates changes in the diagnostic distribution of pediatric ED visits by age group before and after the COVID-19 pandemic. A visual summary of these diagnostic trends is provided in Supplementary Figure S1. The diagnostic distribution of pediatric ED visits demonstrated notable shifts between the pre- and post-COVID-19 periods in all age groups (*p* < 0.001).

In infants younger than 12 months, the proportion of visits for non-specific symptoms and signs (R codes) increased markedly from 42.4% before the pandemic to 63.6% after it. Conversely, respiratory diseases (J codes) and infectious/parasitic diseases (A & B codes) decreased significantly (J: 21.6% to 5.8%, A & B: 12.4% to 2.4%). At the same time, the proportion of trauma-related visits (S & T codes) doubled from 5.1% to 11.2%.

Among children aged 1–6 years, R-code-related visits increased from 37.4% to 59.6%, J and A & B codes decreased (J: 24.6% to 5.7%; A & B: 9.4% to 1.6%), and trauma-related visits (S & T) increased from 15.7% to 22.5%.

In the 7–12-years group, R code visits increased from 27.6% to 47.2%, infectious-disease-related visits declined (J: 14.4% to 6.6%, A & B: 9.0% to 1.7%), and the proportion of S & T-related visits remained relatively stable (28.1% to 27.3%).

For adolescents, aged 13–18 years, visits for symptoms and signs (R codes) increased from 28.6% to 45.4%, J and A & B code visits declined (J: 9.7% to 6.6%, A & B: 10.0% to 2.7%), and trauma-related visits (S & T) decreased slightly (30.0% to 25.5%).

After the onset of COVID-19, visits for respiratory infections (J codes) and infectious diseases (A & B codes) declined significantly across all age groups, whereas visits related to symptoms and signs (R codes) showed a marked increase.

Injury-related visits (S & T) increased, particularly among infants and young children (<6 years), whereas adolescents (13–18 years) showed a slight decline in trauma-related visits after the pandemic began.

### 3.3. Age-Specific Shifts in KTAS Acuity Levels

Table 5 compares the KTAS distribution across different age groups before and after the COVID-19 pandemic. A visual comparison of the changes across age groups is available in Supplementary Figure S2.

Among infants younger than 12 months, the proportions across KTAS levels remained largely stable, with KTAS 3 being the most common in both periods. After the COVID-19 pandemic, the proportion of KTAS 3 increased from 44.6% to 49.7%, while KTAS 2 decreased from 22.1% to 18.8%. The proportion of KTAS 1 remained low and relatively unchanged (0.5% to 0.8%, *p* = 0.550). Minor increases were observed in KTAS 3 and KTAS 4, and the KTAS 2 and KTAS 5 proportions decreased slightly.

The 1–6-years age group had a notable increase in low-acuity visits. The proportion of KTAS 4 increased from 45.4% before the pandemic to 52.9% after, and KTAS 3 decreased from 43.9% to 37.9%. As for the high-acuity categories (KTAS 1 and 2), there was no statistically significant change in KTAS 1 (0.3% to 0.7%, *p* = 0.209), and KTAS 2 (2.7% to 2.4%) decreased slightly.

Among children aged 7–12 years, the distribution shifted toward lower acuity. KTAS 4 increased slightly from 71.9% to 74.0%, and KTAS 3 decreased from 18.0% to 16.9%. KTAS 1 and 2 remained low in both periods.

For adolescents (13–18 years), the pattern was similar. KTAS 3 increased from 17.4% to 22.5%, and KTAS 4 decreased slightly from 67.1% to 65.6%. KTAS 5 visits decreased from 12.0% to 9.4%, suggesting a relative shift toward moderate-acuity presentations in this group.

Overall, most age groups, especially younger children (1–12 years), saw a mild trend toward an increased proportion of lower-acuity cases (KTAS 4) after the pandemic, though the older age groups (13–18 years) exhibited more varied patterns, including a relative rise in moderate-acuity (KTAS 3) visits. KTAS 1–2 (high acuity) visits remained relatively stable across all age groups. KTAS 3 (moderate acuity) increased slightly in infants and adolescents, and KTAS 4 (lower acuity) showed a notable increase in children aged 1–6 years. The proportion of KTAS 5 (non-urgent) visits declined in most age groups after the pandemic.

## 4. Discussion

Our analysis of eight years of pediatric ED visits at Samsung Changwon Hospital reveals significant shifts in utilization patterns, patient characteristics, triage levels, and diagnostic distributions before and during the COVID-19 pandemic, highlighting how the pandemic affected various aspects of pediatric emergency care in Korea. In our center, the number of pediatric emergency room patients gradually increased in 2021–2023 as pandemic restrictions eased, but it remained below pre-COVID-19 levels until the end of the study period, reflecting a slow and incomplete recovery pattern across the country for three years [11]. The persistent decline of pediatric ED visits shows how significantly the behavior of caregivers seeking medical service changed during the pandemic in Korea, where caregivers have traditionally sought rapid ED care for their children.

The demographic changes observed are age-specific shifts in healthcare utilization patterns. As the overall number of pediatric ED visits decreased following the onset of COVID-19, the proportion of children younger than 6 years old especially decreased. This change could reflect a combination of factors, including parental concerns about potential exposure to COVID-19 in the hospital, particularly of young children, and a reduced incidence of infectious diseases due to school closures, widespread mask use, and improved hygiene practices during the pandemic period [12,13,14]. The changes in acuity distribution and outcomes present a complex picture. The increase in lower-acuity visits (KTAS 4), accompanied by higher discharge rates, might suggest that children with mild illnesses visited the ED more often during the pandemic. Several factors have been suggested to explain why patients with mild symptoms visit the ED, including the unavailability of primary care services, overestimation of symptom severity by non-medical caregivers, and excessive reliance on tertiary hospital care [15,16]. Conversely, the slight rise in the highest-acuity cases (KTAS 1) and increased ambulance use suggest that some patients delayed a trip to the hospital until their conditions worsened. One notable finding is a post-pandemic increase in body weight among younger children presenting to the ED.

Our study showed significant weight increases among children aged 3–4, 7–8, 9–10, 11–12, and 13–14 years in the post-COVID-19-pandemic period. This suggests that younger school-aged children and early adolescents were particularly vulnerable to weight gain during the pandemic. Such increases are likely attributable to extended time indoors, reduced physical activity, disrupted routines, and dietary changes during lockdowns [17]. In Korea, the prevalence of childhood obesity has risen sharply since the onset of COVID-19, with national data showing an increase from approximately 15% in 2019 to 19% in 2021 [18], and smaller clinical studies reporting significant increases in body mass index (BMI) z-scores among children during periods of social distancing [19]. Similar trends were observed in the United States, where a longitudinal Centers for Disease Control and Prevention (CDC) study found that the rate of BMI increase nearly doubled during the pandemic, compared with the pre-pandemic years, with the most pronounced changes seen in younger school-aged children and those who had previously been of healthy weight [17]. The elevated body weight observed among young school-aged children and early adolescents during the pandemic may have significant long-term consequences. Obesity beginning in childhood often persists into adulthood and has been linked to a substantially increased risk of lifestyle-related conditions such as type 2 diabetes, hypertension, dyslipidemia, and cardiovascular disease, all of which contribute to premature morbidity and mortality [20].

A significant reduction in pediatric ED visits for respiratory and infectious diseases was observed as one of the most prominent effects of the COVID-19 pandemic. In the pre-2020 period, respiratory infections (ICD-10 J00–J99, e.g., bronchiolitis, pneumonia) and febrile infectious illnesses (ICD-10 A00–B99) were among the most common reasons for pediatric ED visits. This shifted dramatically after COVID-19 pandemic control policies (wearing masks, social distancing, school closures) were in place. We observed a significant decline in ED visits for respiratory illnesses and communicable infections in 2020 and the subsequent years. During the period of strict public health interventions, the incidence of common pediatric viral infections in Korea dropped to levels far below normal seasonal variation, with many routine viral illnesses nearly disappearing. National infectious disease surveillance data confirm that non-pharmaceutical interventions led to an unprecedented reduction in these infections; for example, in 2020, the incidence of respiratory viral infections in children decreased to about 20% of the usual level [21]. Other countries similarly reported that pediatric clinic visits for respiratory infections dropped by more than half during lockdowns and that influenza and respiratory syncytial virus (RSV) seasons were virtually absent in 2020–2021 [22,23]. The most likely explanation is that public health policies prevented the spread of routine viruses because children had reduced contact in schools and public spaces. Additionally, heightened hygiene and mask-wearing would have suppressed the transmission of not only COVID-19 but also other pathogens [24]. In contrast to infections, the pattern of injury-related ED visits during the pandemic followed a more complex trajectory, with notable age-specific differences. Overall, we found that the absolute number of pediatric trauma visits declined in 2020 (consistent with the general drop in ED attendance), but injuries composed a greater proportion of our ED visit volume after COVID-19 began. In younger children, especially those younger than school-age, injury-related ED visits increased relative to pre-pandemic years, whereas in adolescents, we observed a decrease in injury-related cases. These divergent trends likely reflect changes in children’s daily environments under pandemic conditions. With restrictions on schools, daycares, and outdoor activities, younger children spent more time at home, which led to an increase in home-related injuries such as falls, burns, and ingestions. A Korean multi-center study reported that, in 2020, the proportion of pediatric injuries occurring at home rose from 58% to 68%, with a significant increase in fall-related injuries among infants younger than one year [25,26]. Similar patterns were observed internationally; both French and Italian multi-center studies reported that the COVID-19 lockdown substantially increased the proportion of pediatric domestic accidents, particularly falls and upper limb injuries in France and a threefold rise in domestic accident incidence in Italy [27,28]. These findings suggest that prolonged time spent indoors and reduced supervision during lockdowns shifted the burden of injuries from outdoor and sports-related activities to the home environment. Our finding of increased ED trauma visits in toddlers and preschoolers aligns with those observations, suggesting that home became a primary site of injury when curious young children were confined there for long periods. However, among adolescents, a decline in injury-related emergency visits was observed in our study. Consistent with these findings, a previous study observed a significant reduction in pediatric fractures following the onset of COVID-19, alongside a shift in injury patterns: home-related injuries increased from 32.5% to 57.8%, but sports- and playground-related injuries declined markedly [29]. These trends suggest that the cancellation of organized sports and interactive physical activities and the closure of playgrounds during the pandemic likely contributed to a decrease in the minor trauma cases typically seen in pediatric EDs, due to fewer opportunities for outdoor and recreational injuries to occur [29,30].

Beyond these overall patterns, trauma and injury diagnoses also demonstrated distinct age-specific shifts. Head-injury-related visits increased consistently across all age groups. In preschool children, foreign body ingestion and elbow injuries remained common, likely associated with increased time at home and reduced supervision. However, among adolescents, new patterns emerged, with some categories of minor injuries declining, whereas ankle sprains (S93.49) and particularly drug-related injuries (T42.7) became more prominent in the post-pandemic period. Our finding of increased drug-related injuries is consistent with international evidence. A Canadian pediatric ED study reported that the largest proportional increase in poisonings occurred in teenagers, primarily due to intentional self-harm and recreational drug use, with cannabis- and vaping-related presentations rising markedly during the pandemic [31]. Similarly, a U.S. national study found that prescription-opioid overdose ED visits, which had been declining through 2019, rose substantially in 2020 among adolescents aged 12–17 years, a surge attributed to pandemic-related factors such as worsening mental health, reduced access to prescription opioids, and the increased availability of illicit synthetic opioids [32]. Overall, these findings demonstrate that the pandemic not only altered the epidemiology of pediatric emergency department visits but also increased the risk of substance misuse among adolescents, highlighting the need for age-specific injury prevention and integrated mental health support.

When examining acuity distributions, we found notable age-specific shifts in KTAS levels after the COVID-19 pandemic. Previous studies showed that non-urgent ED visits decreased during the COVID-19 pandemic [33,34], but we found that after the COVID-19 pandemic, KTAS distributions showed a modest shift toward lower-acuity presentations, particularly among younger children. Among infants, KTAS 3 visits increased from 44.6% to 49.7%, while KTAS 2 decreased. In the 1–6-years age group, KTAS 4 visits increased notably (45.4% to 52.9%), whereas KTAS 3 and 5 visits decreased. This trend might reflect increased parental concern about mild symptoms and reduced access to outpatient services during the pandemic, which likely led to the redirection of minor procedures and non-urgent consultations to the pediatric ED, thereby increasing the proportion of low-acuity cases [15]. School-aged children (7–12 years) demonstrated relatively stable patterns, whereas adolescents showed a relative rise in KTAS 3 visits (17.4% to 22.5%), possibly due to delayed care-seeking during the pandemic. A prior study found that about one in five caregivers delayed visiting the ED due to concerns about hospital-acquired infections, with many reporting symptom worsening, and that might have contributed to the observed increase in moderate-acuity cases [35]. High-acuity visits (KTAS 1–2) remained relatively stable across all age groups, suggesting that emergency services continued to be accessed appropriately for critical cases. These findings underscore the complex impact of the pandemic on triage patterns, particularly for KTAS 3, which remains the most challenging category in pediatric emergency care.

Our study has several limitations. First, as this is a single-center study, it is difficult to clearly attribute all observed changes to the pandemic, and the findings may not be generalizable to other regions or healthcare settings. Second, the retrospective design may cause information bias and does not allow for control of potential confounding factors such as public health policies and caregiver behaviors during the pandemic. Third, classification based on KTAS and ICD-10 codes may be subject to inter-observer variability or misclassification. Fourth, the absence of population-based denominators restricts our ability to estimate true incidence rates, and our results should be interpreted as trends in ED utilization rather than population-level changes. Fifth, socioeconomic variables were not available in our dataset. Factors such as household income, parental education, and living environment may have influenced patterns of emergency department utilization and trauma risk during the pandemic. Finally, although our dataset spans eight years and covers both pre-pandemic and immediate post-pandemic periods (2016–2023), longer-term follow-up will be required to determine whether the observed changes represent temporary disruptions or more persistent shifts in pediatric emergency utilization. To address these limitations, future studies should conduct multi-center or population-based designs using age-specific denominators to improve generalizability and enable incidence-based comparisons. A prospective design may allow for standardized data collection and reduce misclassification bias, thereby enhancing the validity of the findings.

## 5. Conclusions

In conclusion, this analysis provides an eight-year overview of how the COVID-19 pandemic changed pediatric ED utilization in Korea. We observed a marked decline in overall visits, shifts in the types of illnesses seen, and various effects across age groups. The study’s strengths, including its long-term range and detailed age and diagnosis data, enabled us to uncover meaningful trends, such as increased body weight in young children and age-specific diagnosis and severity trends. Importantly, we identified concerning trends such as the increased body weight in younger children and rising drug-related injuries in adolescents, both of which may have long-term public health implications. These insights will guide the direction of emergency preparedness and public health policy, ensuring the continuity of essential pediatric emergency services during crises, addressing indirect health effects, and adapting interventions to the needs of different pediatric age groups. Based on the lessons learned from this period, healthcare systems can better plan for future pandemics or other disruptive situations to ensure that they can maintain a high standard of care for children under all conditions.

## Figures and Tables

**Table 1:** Demographic and clinical characteristics of pediatric emergency department visits (*N* = 71,560).

	Before COVID-19 (2016–2019)	After COVID-19 (2020–2023)	*p*-Value
*N*	49,526	22,034	
Sex			<0.001
Female	21,764 (43.94)	9368 (42.52)	
Male	27,762 (56.06)	12,666 (57.48)	
Age			<0.001
<12 months	9873 (19.93)	2785 (12.64)	
1–6 years	28,838 (58.23)	11,259 (51.10)	
7–12 years	6164 (12.45)	4215 (19.13)	
13–18 years	4651 (9.39)	3775 (17.13)	
KTAS (*n* = 60,792)			<0.001
1	166 (0.43)	161 (0.74)	
2	2415 (6.21)	881 (4.03)	
3	14,905 (38.31)	7131 (32.59)	
4	18,275 (46.97)	12,221 (55.85)	
5	3150 (8.10)	1487 (6.80)	
Outcome			<0.001
Discharge home	40,359 (81.49)	19,040 (86.41)	
Admission	9038 (18.25)	2851 (12.94)	
Transfer	99 (0.20)	113 (0.51)	
Death	30 (0.06)	30 (0.14)	
Ambulance transport (*n* = 34,196)			<0.001
No	11,564 (93.90)	19,626 (89.69)	
Yes	751 (6.10)	2255 (10.31)	
Day of week			<0.001
Weekdays	25,375 (51.24)	12,038 (54.63)	
Weekend + Holiday	24,151 (48.76)	9996 (45.37)	
Arrival time			0.084
Day time (06:00~18:00)	18,699 (37.76)	8170 (37.08)	
Night time (18:00~06:00)	30,827 (62.24)	13,864 (62.92)	
Influenza (*n* = 15,089)			<0.001
Negative	8121 (78.87)	4084 (85.23)	
Influenza A	1528 (14.84)	641 (13.38)	
Influenza B	634 (6.16)	66 (1.38)	
Influenza A & B (Both)	14 (0.14)	1 (0.02)	
COVID-19 (*n* = 9351)			-
Negative	-	8568 (91.63)	
Positive	-	738 (7.89)	
Borderline	-	45 (0.48)	
Vital signs			
Body temperature (*n* = 64,608)	37.71 ± 1.13	37.57 ± 1.17	<0.001
SpO_2_ (*n* = 48,037)	98.29 ± 3.60	98.24 ± 2.75	0.079
RR (*n* = 64,477)	27.64 ± 6.82	23.75 ± 5.57	<0.001
<12 months	35.12 ± 6.54	30.85 ± 6.02	<0.001
1–6 years	27.72 ± 5.01	24.81 ± 4.43	<0.001
7–12 years	22.41 ± 3.86	21.14 ± 3.30	<0.001
13–18 years	19.06 ± 3.25	18.48 ± 3.17	<0.001
Body weight (*n* = 54,704)	19.81 ± 16.28	26.48 ± 20.35	<0.001
<12 months	7.69 ± 3.37	7.69 ± 2.30	0.989
1–2 years	12.10 ± 3.72	12.20 ± 2.97	0.075
3–4 years	16.28 ± 3.56	16.51 ± 2.80	0.001
5–6 years	21.39 ± 4.62	21.39 ± 4.60	0.975
7–8 years	28.12 ± 7.06	28.80 ± 7.42	0.006
9–10 years	36.81 ± 9.40	38.42 ± 10.36	<0.001
11–12 years	46.32 ± 11.57	49.24 ± 12.54	<0.001
13–14 years	55.56 ± 12.85	57.68 ± 13.75	<0.001
15–16 years	62.48 ± 14.05	61.88 ± 14.66	0.349
17–18 years	63.58 ± 14.56	63.91 ± 14.84	0.608

Note: Values are presented as mean ± standard deviation or *n*. KTAS = Korean Triage and Acuity Scale; SpO_2_ = peripheral capillary oxygen saturation; RR = respiratory rate. The number of observations (*N*) varies across variables due to data availability. Body weight and respiratory rate are additionally stratified by age groups to highlight post-pandemic differences.

**Table 2:** Diagnostic distribution of pediatric emergency department visits.

	Total	Before COVID-19 (2016~2019) *n* (%)	After COVID-19 (2020~2023) *n* (%)	*p*-Value
*N*	71,560	49,526	22,034	
Diagnosis				<0.001
R	30,189 (42.19)	18,008 (36.36)	12,181 (55.28)	
S & T	13,109 (18.32)	8158 (16.47)	4951 (22.47)	
J	11,894 (16.62)	10,564 (21.33)	1330 (6.04)	
A & B	5367 (7.50)	4943 (9.98)	424 (1.92)	
L	2733 (3.82)	1860 (3.76)	873 (3.96)	
K	2309 (3.23)	1795 (3.62)	514 (2.33)	
N	1567 (2.19)	1071 (2.16)	496 (2.25)	
H	1198 (1.67)	965 (1.95)	233 (1.06)	
I	683 (0.95)	522 (1.05)	161 (0.73)	
M	566 (0.79)	349 (0.70)	217 (0.98)	
G	550 (0.77)	415 (0.84)	135 (0.61)	
P	535 (0.75)	430 (0.87)	105 (0.48)	
Z	397 (0.55)	157 (0.32)	240 (1.09)	
D	137 (0.19)	103 (0.21)	34 (0.15)	
U	98 (0.14)	0 (0.00)	98 (0.44)	
E	85 (0.12)	60 (0.12)	25 (0.11)	
Q	84 (0.12)	75 (0.15)	9 (0.04)	
F	36 (0.05)	33 (0.07)	3 (0.01)	
O	16 (0.02)	13 (0.03)	3 (0.01)	
C	7 (0.01)	5 (0.01)	2 (0.01)	

Note: Values are presented as *n* (%). Diagnostic categories are based on the International Classification of Diseases, 10th Revision (ICD-10): R = symptoms, signs, and abnormal clinical and laboratory findings; S & T = injury, poisoning, and certain other consequences of external causes; J = diseases of the respiratory system; A & B = certain infectious and parasitic diseases; L = diseases of the skin and subcutaneous tissue; K = diseases of the digestive system; N = diseases of the genitourinary system; H = diseases of the eye, adnexa, ear, and mastoid process; I = diseases of the circulatory system; M = diseases of the musculoskeletal system and connective tissue; G = diseases of the nervous system; P = certain conditions originating in the perinatal period; Z = factors influencing health status and contact with health services; D = diseases of the blood and blood-forming organs; U = codes for special purposes (including COVID-19); E = endocrine, nutritional, and metabolic diseases; Q = congenital malformations, deformations, and chromosomal abnormalities; F = mental and behavioral disorders; O = pregnancy, childbirth, and the puerperium; C = neoplasms.

**Table 3:** Comparison of medical-disease- and trauma-related pediatric emergency department visits.

	Total	Before COVID-19 (2016~2019) *n* (%)	After COVID-19 (2020~2023) *n* (%)	*p*-Value
*N*	71,560	49,526	22,034	<0.001
Medical disease	58,451 (81.68)	41,368 (83.53)	17,083 (77.53)	
Trauma, injury, or poisoning	13,109 (18.32)	8158 (16.47)	4951 (22.47)	

Note: Values are presented as number of cases (*n*) along with percentages (%) of the total pediatric emergency department visits in each period.

**Table 4:** Diagnostic distribution of pediatric emergency department visits by age group before and after the COVID-19 pandemic.

Age/ICD-10	Total	Before COVID-19 (2016~2019) *n* (%)	After COVID-19 (2020~2023) *n* (%)	*p*-Value
<12 months	12,658	9873	2785	<0.001
R	5957 (47.06)	4186 (42.40)	1771 (63.59)	
J	2297 (18.15)	2136 (21.63)	161 (5.78)	
A & B	1295 (10.23)	1228 (12.44)	67 (2.41)	
S & T	816 (6.45)	505 (5.11)	311 (11.17)	
Others	2293 (18.12)	1818 (18.41)	475 (17.06)	
1–6 years	40,097	28,838	11,259	<0.001
R	17,499 (43.64)	10,792 (37.42)	6707 (59.57)	
J	7730 (19.28)	7087 (24.58)	643 (5.71)	
A & B	2879 (7.18)	2698 (9.36)	181 (1.61)	
S & T	7055 (17.59)	4527 (15.70)	2528 (22.45)	
Others	4934 (12.31)	3734 (12.95)	1200 (10.66)	
7–12 years	10,379	6164	4215	<0.001
R	3689 (35.54)	1698 (27.55)	1991 (47.24)	
J	1168 (11.25)	890 (14.44)	278 (6.60)	
A & B	625 (6.02)	552 (8.96)	73 (1.73)	
S & T	2880 (27.75)	1729 (28.05)	1151 (27.31)	
Others	2017 (19.43)	1295 (21.01)	722 (17.13)	
13–18 years	8426	4651	3775	<0.001
R	3044 (36.13)	1332 (28.64)	1712 (45.35)	
J	699 (8.30)	451 (9.70)	248 (6.57)	
A & B	568 (6.74)	465 (10.00)	103 (2.73)	
S & T	2358 (27.98)	1397 (30.04)	961 (25.46)	
Others	1757 (20.85)	1006 (21.63)	751 (19.89)	

Note: Values are presented as number of cases (*n*) along with percentages (%) within each age group. Percentages were calculated relative to the total number of visits in the corresponding age group. “Others” includes all remaining ICD-10 categories not listed separately. ICD-10 category abbreviations (R, J, A & B, S & T) were defined in Table 2.

**Table 5:** Distribution of Korean Triage and Acuity Scale (KTAS) scores among pediatric emergency department visits by age group before and after the COVID-19 pandemic.

Age Group	KTAS Level	Before COVID-19 *n* (%)	After COVID-19 *n* (%)	*p-*Value
<12 months	1	37 (0.5%)	21 (0.8%)	0.550
	2	1610 (22.1%)	496 (18.8%)	<0.001
	3	3242 (44.6%)	1309 (49.7%)	<0.001
	4	1824 (25.1%)	663 (25.2%)	<0.001
	5	556 (7.6%)	145 (5.5%)	<0.001
1–6 years	1	79 (0.3%)	77 (0.7%)	0.209
	2	626 (2.7%)	271 (2.4%)	<0.001
	3	10,150 (43.9%)	4261 (37.8%)	<0.001
	4	10,494 (45.4%)	5961 (52.9%)	<0.001
	5	1771 (7.7%)	689 (6.1%)	<0.001
7–12 years	1	28 (0.6%)	40 (0.9%)	0.243
	2	76 (1.5%)	43 (1.0%)	0.479
	3	888 (18.0%)	712 (16.9%)	<0.001
	4	3541 (71.9%)	3119 (74.0%)	<0.001
	5	390 (7.9%)	299 (7.1%)	<0.001
13–18 years	1	22 (0.6%)	23 (0.6%)	0.801
	2	103 (2.9%)	71 (1.9%)	0.176
	3	625 (17.4%)	849 (22.5%)	<0.001
	4	2416 (67.1%)	2478 (65.6%)	<0.001
	5	433 (12.0%)	354 (9.4%)	<0.001

Note: Values are presented as number of cases (*n*) along with percentages (%) within each age group. Percentages were calculated relative to the total visits in the corresponding age group. KTAS ranges from level 1 (most urgent) to level 5 (least urgent). *p*-values indicate statistical differences between the pre- and post-pandemic periods.

## Data Availability

The data presented in this study are available on request from the corresponding author. The data are not publicly available due to privacy or ethical restrictions.

## Supplementary Materials

The following supporting information can be downloaded at: https://www.mdpi.com/article/10.3390/children12091232/s1, Figure S1: Diagnostic distribution of pediatric emergency department visits by age group before and after the COVID-19 pandemic; Figure S2: Distribution of Korean Triage and Acuity Scale (KTAS) scores among pediatric emergency department visits before and after the COVID-19 pandemic, stratified by age group; Table S1: Age-specific changes in trauma and injury patterns before and after the COVID-19 pandemic.
