# Supplementary material for: Examining Pediatric Emergency Utilization Trends Before and After the COVID-19 Pandemic: An Eight-Year Cohort Study from a South Korean Tertiary Center
Source: Children (Basel). 2025 Sep 15;12(9):1232. doi: 10.3390/children12091232 (PMC12468405; doi:10.3390/children12091232)

Supplementary Figure S1. Diagnostic distribution of pediatric emergency department visits by age group before and after the COVID-19 pandemic.

The figure compares the proportions of major diagnostic categories — symptoms/signs (R), respiratory diseases (J), infectious diseases (A & B), injuries/trauma (S & T), and others — across four pediatric age groups:

(A) <12 months, (B) 1–6 years, (C) 7–12 years, and (D) 13–18 years.

White bars represent the distribution before COVID-19 (2016–2019), and black bars represent the distribution after COVID-19 (2020–2023).

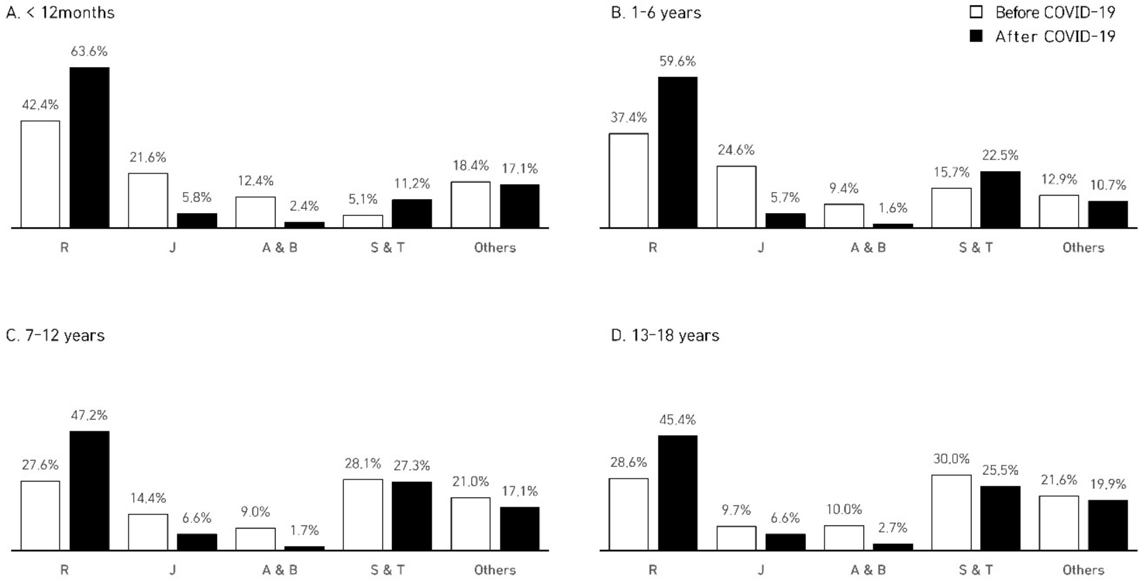

Supplement: Supplementary file 1 [file children-12-01232-s001.zip › children-3814337 supplemental Figure1.pdf]
