# Supplementary material for: Examining Pediatric Emergency Utilization Trends Before and After the COVID-19 Pandemic: An Eight-Year Cohort Study from a South Korean Tertiary Center
Source: Children (Basel). 2025 Sep 15;12(9):1232. doi: 10.3390/children12091232 (PMC12468405; doi:10.3390/children12091232)

Supplementary Figure S2. Distribution of Korean Triage and Acuity Scale (KTAS) scores among pediatric emergency department visits before and after the COVID-19 pandemic, stratified by age group.

White bars represent data from the pre-COVID period (2016–2019), and black bars represent the post-COVID period (2020–2023).

Four pediatric age groups are shown:

(A) <12 months, (B) 1–6 years, (C) 7–12 years, and (D) 13–18 years.

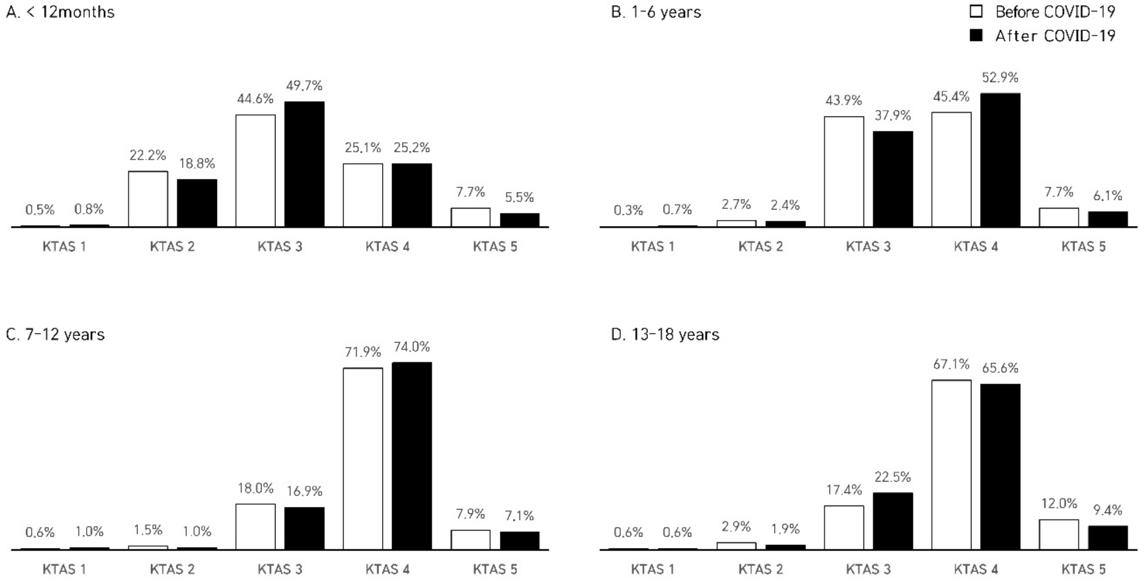

Supplement: Supplementary file 1 [file children-12-01232-s001.zip › children-3814337 supplemental Figure2.pdf]
