# Supplementary material for: Examining Pediatric Emergency Utilization Trends Before and After the COVID-19 Pandemic: An Eight-Year Cohort Study from a South Korean Tertiary Center
Source: Children (Basel). 2025 Sep 15;12(9):1232. doi: 10.3390/children12091232 (PMC12468405; doi:10.3390/children12091232)
Supplement: Supplementary file 1 [file children-12-01232-s001.zip › children-3814337 Table S1.pdf]

Table S1. Age-specific changes in trauma and injury patterns before and after the COVID-19 pandemic

Age	Before COVID		After COVID	
Rank	ICD-10	n (%)	ICD-10	n (%)
<12month				
1	S06.00	113 (22.60)	S06.00	121 (38.91)
2	S53.0	45 (9.00)	S53.0	24 (7.72)
3	S53.19	24 (4.80)	S02.00	11 (3.54)
4	T18.9	20 (4.00)	S61.0A	9 (2.89)
5	T18.9A	19 (3.80)	S06.30	7 (2.25)
1-6 years				
1	S06.00	483 (10.70)	S06.00	487 (9.27)
2	T17.21	415 (9.19)	S53.0	227 (8.98)
3	S53.0	334 (7.40)	T17.1	155 (6.13)
4	S01.0A	285 (6.31)	S01.0A	148 (5.86)
5	T17.1	277 (6.14)	T17.2	139 (5.50)
7-12 years				
1	S06.00	238 (13.89)	S06.00	257 (22.35)
2	T17.21	155 (9.04)	T17.2	108 (9.39)
3	S01.0A	93 (5.43)	T17.21	37 (3.22)
4	S01.8A	72 (4.20)	S01.0A	32 (2.78)
5	T17.29	50 (2.92)	S60.0A	30 (2.61)
13-18 years				
1	S06.00	168 (12.27)	S06.00	134 (13.96)
2	T17.21	83 (6.06)	T17.2	51 (5.31)
3	S01.8A	64 (4.67)	S93.49	48 (5.00)
4	S93.49	43 (3.14)	T42.7	37 (3.85)
5	S02.20	40 (2.92)	S61.0A	30 (3.13)

Note: Values are presented as number of cases (n) with percentages (%) within each age group, calculated relative to the total trauma and injury visits in that group. Only the five most frequent diagnoses are shown for each age category. Diagnoses are coded according to the *International Classification of Diseases, 10th Revision (ICD-10)*. Some codes represent extensions from the *Korean Standard Classification of Diseases (KCD)*, a national adaptation of ICD-10.

ICD-10/KCD codes: S06.00, concussion *(KCD extension)*; S53.0, dislocation of radial head; S53.19, other sprain and strain of elbow *(KCD extension)*; T18.9/T18.9A, foreign body in alimentary tract, unspecified *(A = KCD extension)*; S61.0A, laceration of finger *(KCD extension)*; S06.30, focal brain injury without open intracranial wound *(KCD extension)*; T17.21, foreign body in pharynx, food *(KCD extension)*; T17.1, foreign body in nostril; T17.2, foreign body in pharynx; T17.29, foreign body in pharynx, unspecified *(KCD extension)*; S01.0A, laceration of scalp *(KCD extension)*; S01.8A, laceration of face *(KCD extension)*; S60.0A, contusion of finger *(KCD extension)*; S93.49, sprain and strain of ankle, part unspecified; T42.7, poisoning by antiepileptic and sedative-hypnotic drugs, unspecified; S02.20, fracture of nasal bone, closed *(KCD extension)*.
